# Comprehensive assessment and prediction of calcium-phosphorus precipitation in parenteral nutrition using advanced monitoring and data mining techniques

**DOI:** 10.3389/fnut.2026.1739220

**Published:** 2026-07-09

**Authors:** Qian Xu, Gen-yu Xie, Fan Xu

**Affiliations:** 1 Graduate School, Kunming Medical University, Kunming, China; 2 920th Hospital of People's Liberation Army Joint Logistic Support Force, Kunming, China

**Keywords:** calcium-phosphorus compatibility, data mining, parenteral nutrition safety, predictive modeling, total nutrient admixture

## Abstract

**Background:**

Calcium-phosphate precipitation poses a significant risk in parenteral nutrition. Existing compatibility studies are predominantly qualitative with inconsistent safety thresholds. This study aimed to develop a quantitative predictive framework to overcome these limitations.

**Methods:**

A novel high-sensitivity real-time precipitation monitoring device was developed. The compatibility of six calcium-phosphorus formulation pairs was systematically evaluated under clinically relevant conditions using a factorial design, generating 15,342 experimental observations. Four data mining models—C5.1, CHAID, Support Vector Machine, and Artificial Neural Network—were constructed and compared.

**Results:**

Analysis established distinct compatibility profiles. Precipitation was exclusive to inorganic phosphate combinations, specifically calcium chloride or calcium gluconate with potassium dihydrogen phosphate, while organic phosphate formulations remained stable within the tested concentration ranges. Six significant predictors were identified: calcium concentration, phosphorus concentration, pH, and temperature as risk factors, alongside 18AA-II and 18AA-V as protective factors. The C5.1 algorithm demonstrated optimal performance, achieving 98.5% predictive accuracy. External validation using 100 independent clinical samples confirmed robust performance (95% accuracy), facilitating the creation of a web-based clinical decision platform.

**Conclusions:**

This study provides a systematic quantitative evaluation of clinically used calcium-phosphorus combinations under clinically relevant conditions. A data-driven framework that links experimental evidence with clinical decision-making and contributes to improved parenteral nutrition safety is established by integrating real-time precipitation monitoring, a large experimental dataset, predictive modeling, and external validation.

## 1 Introduction

Total Nutrient Admixture (TNA), comprising macronutrients (dextrose, amino acids, and intravenous lipid emulsion) combined with electrolytes, vitamins, minerals, trace elements, and sterile water in an intravenous solution (1), serves as a critical nutritional intervention when oral or enteral nutrition is contraindicated or inadequate. However, TNA presents significant risks as the components may interfere with each other's stability and even generate harmful substances. One major concern is the precipitation of calcium (Ca) and phosphorus (P), which is a serious issue in TNA due to its potential to cause severe complications, such as pulmonary embolism, which can result in patient death (2–5). Nevertheless, calcium and phosphorus are essential components of total parenteral nutrition (6, 7). Calcium plays a crucial role in information transfer and substance metabolism, while phosphorus is vital for bone formation and oxidative phosphorylation for Adenosine triphosphate (ATP) production (8, 9). Therefore, an in-depth study of calcium and phosphorus compatibility in TNA is essential.

In recent years, a number of studies have been conducted in the field of calcium-phosphorus compatibility in parenteral nutrition (10–13). However, the majority of this research remains qualitative in nature. While these investigations have successfully identified the risk of precipitation in calcium-phosphorus formulations and highlighted contributing factors such as pH, amino acid profiles and concentrations, and specific calcium/phosphate salts, they have failed to establish quantitative dose-response relationships or determine the relative impact of each variable.

Several attempts to quantify these relationships have been hampered by multifactorial challenges: the complexity of parenteral nutrition solutions, inadequate sample sizes for detecting precipitation events, and limitations in analytical sensitivity. Consequently, no universal compatibility model or definitive concentration ranges for calcium and phosphate formulations have been established. The literature reveals significant discrepancies between studies—for instance, Bouchoud et al. (14) and Newton and Driscoll (15) reported conflicting precipitation thresholds for similar formulations, while Prinzivalli and Ceccarelli's (16) proposed *K* value (the product of calcium and phosphate ion concentrations) shows poor generalizability across different studies. For calcium chloride with disodium hydrogen phosphate, reported compatible *K* values range from 4.5 to 21 vs. 5.19–6.74; for glucose-1-phosphate with calcium ions, the discrepancy is even more pronounced (>1,250 vs. 623.7–1,100.3). These inconsistencies prevent definitive conclusions beyond qualitative observations that calcium gluconate with sodium glycerophosphate appears safest.

These limitations stem from fundamental methodological challenges. The complex baseline environment of parenteral nutrition solutions—where pH, temperature, amino acids, electrolytes, and carbohydrates interact—requires substantial experimental investment when using traditional approaches. Conventional factorial designs employing concentration increments fail to adequately characterize the compatibility landscape, generating insufficient data for robust model development. While the mechanism of calcium-phosphate precipitation is well-understood and analogous to solubility equilibrium studies, the practical assessment requires empirical validation of how specific TNA components interact to influence precipitation under clinically relevant conditions.

To address these limitations, this study aims to develop a novel dynamic precipitation monitoring system to enable real-time detection of calcium-phosphate compatibility. Building upon this technological foundation, factorial design will be employed to generate a comprehensive dataset of compatibility outcomes under clinically relevant conditions. The acquired experimental data will then be utilized to construct predictive models through advanced data mining techniques. Ultimately, this integrated approach seeks to establish a precision forecasting application capable of providing accurate compatibility predictions for diverse clinical formulation scenarios, thereby significantly enhancing the safety of parenteral nutrition therapy.

## 2 Materials and methods

### 2.1 Chemicals and reagents

All reagents used in this study were of analytical grade, and all pharmaceutical preparations were commercially available injectable products. Calcium chloride (CaCl_2_; 99.7% purity) and hydrochloric acid (HCl, 37.1 wt%) were obtained from (Sinopharm Chemical Reagent Co., Ltd. Shanghai, China). Calcium gluconate (C_12_H_22_CaO_14_, CaGlu; 98.0% purity) and sodium hydroxide (NaOH; 99.9% purity) were supplied by (Tianjin Damao Chemical Reagent Factory, Tianjin, China). Potassium dihydrogen phosphate (KH_2_PO_4_; 99.0% purity) was procured from (Dalian Meilun Biotechnology Co., Ltd, Dalian, Liaoning, China). Sodium β-glycerophosphate (C_3_H_7_Na_2_O_6_P, β-GP; 99.0% purity) and D-fructose-1,6-bisphosphate (C_6_H_14_O_12_P_2_, D-fructose-1,6-bisphosphate (FBP); 98.0% purity) were acquired from (Hefei Bomei Biotechnology Co., Ltd. Hefei, Anhui, China).

The following commercial injections were used: calcium chloride injection (Taibang Biological Products Co., Ltd. Tai'an, Shandong, China), calcium gluconate injection (Zhejiang Guojing Pharmaceutical Co., Ltd. Lishui, Zhejiang, China), compound potassium hydrogen phosphate injection (Shanxi Pude Pharmaceutical Co., Ltd. Datong, Shanxi, China), sodium glycerophosphate injection (Fresenius Kabi Huarui Pharmaceutical Co., Ltd. Wuxi, Jiangsu, China), and fructose-1,6-diphosphate sodium injection (Guangdong Hongyuan Pharmaceutical Group Co., Ltd. Dongguan, Guangdong, China). Compound amino acid injections (18AA-II at 8.5% and 11.4%; 18AA-V at 3.2%; all 250 ml) and glucose injections were provided by Sinopharm Yunnan Co., Ltd, Kunming, Yunnan, China. Ultrapure water was produced using a (Merck Millipore, Darmstadt, Germany.) water purification system.

### 2.2 Device materials

Voltage regulator (model: TKR-1500 VA) was purchased from Yueqing Zhengnan Electrical Appliance Co., Ltd. Yueqing, Zhejiang, China. Lasers (power: 150 mw) was purchased from Shenzhen Huashang Laser Technology Co., Ltd. Shenzhen, Guangdong, China. PH thermometer (model: CS 12303) was purchased from Shanghai Zhenmai Instrument Co., Ltd. Shanghai, China. Water bath circulation device and double-layer beaker were purchased from Shanghai Language Union Co., Ltd. Shanghai, China. Electromagnetic stirrer (power: 150 mw) was purchased from Suzhou Jiulian Technology Co., Ltd. Suzhou, Jiangsu, China. Illuminance meter (model: 1010D) was purchased from Shanghai Qidi Industrial Co., Ltd., Shanghai, China.

### 2.3 Dynamic monitoring device based on photoresist method

**Figure 1:**
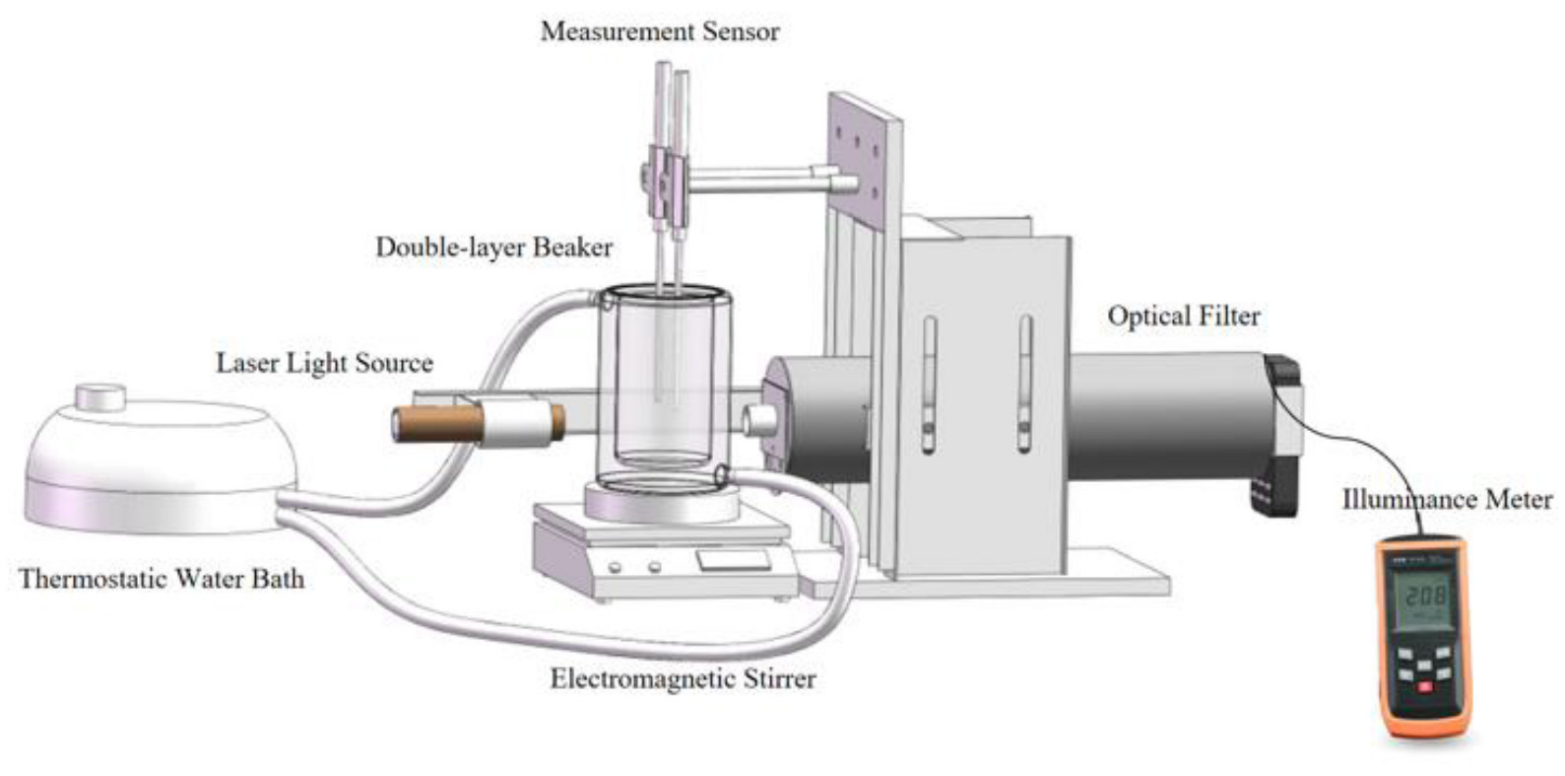
Aqueous-phase dynamic precipitation monitoring device.

A dynamic precipitation monitoring system was developed based on the photoresistance principle for real-time detection of particulate formation. As shown in Figure 1, the system comprised three modules: (1) an illumination module with a stabilized 520-nm laser; (2) a reaction module with a thermostated jacketed beaker, pH/temperature probes, and a stirrer; and (3) a detection module with an illuminance meter and an optical filter to exclude stray light. During operation, phosphorus-containing solutions were placed in the beaker and titrated with calcium solutions. The formation of precipitates quantitatively attenuated the light transmission, which was recorded as a reduction in illuminance, enabling real-time monitoring of precipitation kinetics under simulated physiological conditions.

#### 2.3.1 Methodology establishment

##### 2.3.1.1 Stability

The stability of the monitoring system was systematically evaluated through three critical components: optical configuration, laser intensity calibration, and mixing optimization.

Initially, comprehensive screening of optical components was conducted. Comparative analysis of lasers across different wavelengths (green, red, purple) from multiple manufacturers (Starting Point, Radium Blue) revealed that red and purple lasers induced significant signal instability in illuminance readings. Consequently, green lasers were identified as the optimal wavelength for subsequent investigations. Further evaluation of four green laser sources (FU, Chaonai Technology, Radium Blue, Starting Point) coupled with various illuminance meters (VICTOR 1010D, DT1334A, TES1339, KOMAX) was performed using a standardized setup incorporating voltage stabilization and stray light elimination. The FU brand 520-nm adjustable laser and VICTOR 1010D illuminance meter demonstrated superior performance with stabilization times under 10 min, significantly shorter than the >15 min required by alternative configurations.

Subsequently, laser intensity parameters were optimized through titration experiments. The FU 520-nm laser was operated at adjustable intensities while monitoring illuminance values categorized into discrete ranges ( ≤ 1 × 10^2^ Lux, 2–3 × 10^2^ Lux, 3–5 × 10^2^ Lux, 5–10 × 10^2^ Lux, ≥10 × 10^2^ Lux). Following 30-min baseline stabilization, calcium chloride titrations into KH_2_PO_4_ solutions revealed that initial illuminance values of 2–3 × 10^2^ Lux provided optimal sensitivity, exhibiting consistent 1 × 10^2^ Lux decrements per 1 ml CaCl_2_ addition without sensor saturation or damage. This intensity range was therefore established as standard.

Finally, mixing efficiency was quantified by evaluating electromagnetic stirrer speeds at 500, 1,000, and 1,500 rpm. Maximum rotation at 1,500 rpm effectively minimized vortex-induced bubble formation while ensuring rapid reactant homogenization, consequently enhancing precipitation kinetics measurement reliability.

In summary, systematic standardization established the following optimal parameters: FU 520-nm laser with VICTOR 1010D detector, initial illuminance of 2–3 × 10^2^ Lux, and stirring at 1,500 rpm. This configuration ensured signal stability, measurement precision, and reaction reproducibility throughout all experimental procedures.

##### 2.3.1.2 Sensitivity

The sensitivity of the custom-built monitoring device was assessed against a clinical-standard particle analyzer (GWF-8JA) to ensure that the selected optical threshold was anchored to pharmacopeial safety requirements.

A blank control solution (20 mmol/L KH_2_PO_4_, pH 8.0) was first analyzed to establish the baseline (Δ = 0 Lux). The system was then titrated stepwise with 10 mmol/L CaCl_2_ to generate incremental illuminance reductions ranging from Δ = 1 to 8 × 10^2^ Lux. At each ΔLux level, triplicate 25-ml samples were collected after 30 min of equilibration and analyzed in parallel using the reference instrument.

After correction for baseline values (0.7 particles/ml for insoluble particles ≥10 μm and 0.5 particles/ml for insoluble particles ≥25 μm), a comparison with the Chinese Pharmacopeia(17) and the United States Pharmacopeia (18) standards for insoluble particulate matter in large-volume parenterals demonstrated that an illuminance decrease of Δ = 5 × 10^2^ Lux consistently corresponded to particle counts exceeding the pharmacopeial safety limits for injectable solutions, namely, not more than 25 particles/ml for particles ≥10 μm and not more than 3 particles/ml for particles ≥25 μm (Table 1). This value was therefore adopted as the operational threshold for precipitation in all subsequent experiments. It should be noted that the Chinese Pharmacopeia and the United States Pharmacopeia use the same key particle-size categories, namely ≥10 and ≥25 μm, and the same numerical limits for insoluble particulate matter in large-volume parenterals. Therefore, the optical threshold selected in this study was not specific to a single national pharmacopeial standard, but corresponded to a safety criterion that is directly comparable between these two widely used compendial frameworks.

**Table 1:** Number of insoluble particles in the reaction solution.

Illuminometer value (Lux)	Average number of insoluble particles (per/ml)^a^	
	≧10 μm	≦25 μm
1	1.6	0.2
2	4.9	0
3	17.7	1.1
4	24.7	2.8
5	56.5	2.85
6	173.7	3.05
7	273.3	3.2
8	336.7	3.4

^a^According to the latest editions of the Chinese Pharmacopeia and the United States Pharmacopeia, for intravenous fluids with a labeled volume of 100 ml or more, the number of particles should not exceed 25 per ml for particles ≥10 μm, and should not exceed 3 per ml for particles ≥25 μm, unless otherwise specified.

Across the full concentration gradient, a strong correlation (*R*^2^ = 0.98) was observed between the photometric signal and particle counts. These results indicate that the optical signal provided a reliable quantitative surrogate for precipitation formation within the present experimental system. Accordingly, the threshold used in this study was derived from parallel instrumental calibration and anchored to a pharmacopeial standard rather than being selected empirically.

##### 2.3.1.3 Precision

Method precision was evaluated by measuring calcium chloride consumption at three concentrations of potassium dihydrogen phosphate (10, 15, and 20 mmol L^−1^), with six independent replicates at each level under standardized conditions. Precision was quantified as the relative standard deviation (RSD). The resulting RSD values of 0.18, 0.15, and 0.12 at 10, 15, and 20 mmol L^−1^ KH_2_PO_4_, respectively, all of which were below the acceptable threshold of 0.2, confirm the high reproducibility and reliability of the monitoring system across the tested concentration range.

##### 2.3.1.4 External validation

To further evaluate the reliability and generalizability of the monitoring system, an external validation study was conducted in an independent laboratory operating under Good Manufacturing Practice (GMP) guidelines. The validation protocol was designed according to established principles for analytical instrument comparison and method validation.

A total of thirty standardized calcium-phosphate compatibility samples were analyzed in parallel using the custom-built monitoring device and a GWF-8JA particle analyzer compliant with the requirements of the Chinese Pharmacopeia as the reference method. The developed system is based on the light-obscuration/photoresistance principle combined with dynamic solubility monitoring, enabling continuous real-time detection of precipitation onset and progression. Complete concordance was observed between the two systems, with both methods identifying precipitation formation in 18 of the 30 samples. In this study, the GWF-8JA particle analyzer, a device recommended by the Chinese Pharmacopeia for measuring insoluble particulate matter based on the light obscuration principle, was employed. Compared with the Particle Sizing Systems (PSS) (AccuSizer series) particle analyzer recommended by the United States Pharmacopeia, the GWF-8JA is slightly less capable of detecting large particles in the tail region and performing detailed particle size distribution analysis. However, the accuracy of both instruments for measuring 10 and 25 μm particles is equivalent. Therefore, the GWF-8JA was selected for external validation in this study.

These findings support the reliability and practical applicability of the custom-built monitoring system for detecting subvisible particle formation in parenteral nutrition compatibility studies. The developed system also provides additional value by enabling continuous real-time monitoring of precipitation onset and progression during compatibility assessment. From the perspective of clinical translation, alignment of the operational threshold with the limits for large-volume parenterals specified in both the Chinese Pharmacopeia and the United States Pharmacopeia may improve the interpretability of the results across different pharmacopeial contexts.

### 2.4 Experimental methods

#### 2.4.1 Preparation of phosphorus-containing substrates

Following methodological validation, a full factorial design was adopted in which multiple factors were fully crossed at predefined levels, the specific levels are shown in Table 2. All possible combinations were experimentally tested, enabling a systematic assessment of both the main effects of individual factors and the interaction effects among factors. Phosphorus-containing substrates were prepared according to the experimental matrix using deionized water, glucose solutions (5%, 10% w/v), or amino acid solutions (18AA-II at 8.5%, 11.4% w/v; 18AA-V at 3.2% w/v) as solvent systems.

**Table 2:** Structure of the full factorial design and derivation of the dynamic observations.

Phosphate formulation type	Phosphate concentration (mmol/L)^a^	Calcium formulation type	pH	Temperature (°C)	Solvent^b^	Number of experimental groups
KH_2_PO_4_	5, 10, 20	CaCl_2_	6, 7, 8	20, 30	Deionized water	263
					5% Glucose	468
					10% Glucose	379
					3.2% 18AA-V	344
					8.5% 18AA-II	399
					11.4% 18AA-II	381
		CaGlu			Deionized water	299
					5% Glucose	392
					10% Glucose	392
					3.2% 18AA-V	376
					8.5% 18AA-II	427
					11.4% 18AA-II	398
β-GP	5–150	CaCl_2_			Deionized water	440
					5% Glucose	440
					10% Glucose	440
					3.2% 18AA-V	440
					8.5% 18AA-II	440
					11.4% 18AA-II	440
		CaGlu			Deionized water	440
					5% Glucose	440
					10% Glucose	440
					3.2% 18AA-V	440
					8.5% 18AA-II	440
					11.4% 18AA-II	440
FBP		CaCl_2_			Deionized water	462
					5% Glucose	462
					10% Glucose	462
					3.2% 18AA-V	462
					8.5% 18AA-II	462
					11.4% 18AA-II	462
		CaGlu			Deionized water	462
					5% Glucose	462
					10% Glucose	462
					3.2% 18AA-V	462
					8.5% 18AA-II	462
					11.4% 18AA-II	462

^a^As no precipitation was observed for the organic phosphate formulations under the tested conditions, their concentration range was expanded to eight levels, up to 150 mmol/L.

^b^Amino acid levels were defined according to clinical practice. Specifically, 18AA-II was evaluated at three levels (0, 8.5%, and 11.4%), whereas 18AA-V was evaluated at two levels (0 and 3.2%).

##### 2.4.1.1 Preparation protocol

For the inorganic phosphate substrate, appropriate amounts of KH_2_PO_4_ (0.0680–2.0400 g) were dissolved in the selected solvent system at two temperatures (20 and 30 °C) to prepare solutions at eight concentration levels (5, 10, 20, 40, 80, 100, 120, and 150 mmol/L). The pH of each solution was then adjusted to the target values (6.0, 7.0, and 8.0 ± 0.1) using 0.1 M NaOH or HCl. The organic phosphate compounds, β-GP and FBP, were prepared in a similar manner using the same temperature range, concentration range, and pH range. All solutions were brought to volume in 100 ml volumetric flasks, sterilized by filtration through a 0.22 μm filter, and 60 ml aliquots were aseptically transferred into double-jacketed beakers.

##### 2.4.1.2 Quality control measures

All preparations complied with USP 797 guidelines (18) for sterile pharmaceutical compounding. Additional quality parameters were verified:

- Sterility testing according to membrane filtration method (USP < 71>)
- Endotoxin levels < 0.25 EU/ml (determined by limulus amebocyte lysate test)
- Osmolality range: 280–310 mOsm/kg (measured by freezing point depression)
- Particulate matter compliance with USP < 788> standards
- pH stability maintenance (±0.1 units over 24 h at 4 °C)

Procedures were performed under International Organization for Standardization (ISO) Class 5 cleanroom conditions with continuous environmental monitoring (particle counts < 3,520/m^3^ for ≥0.5 μm; viable organisms < 1 CFU/m^3^). All containers and filtration assemblies were validated for sterility and non-pyrogenicity prior to use.

This comprehensive approach ensured both methodological precision and clinical relevance of the prepared solutions for parenteral nutrition compatibility assessment.

#### 2.4.2 Preparation of calcium solutions

Calcium solutions were prepared at three clinically relevant concentrations (5, 22, and 45 mmol/L) to evaluate concentration-dependent effects on precipitation formation. For the 22 mmol/L CaCl_2_ solution, 0.3234 g of anhydrous CaCl_2_ (MW: 110.98 g/mol) was dissolved in deionized water and quantitatively transferred to a 100 ml volumetric flask. Identical procedures were applied for CaGlu solutions using stoichiometrically equivalent masses. All solutions were brought to volume with deionized water, followed by sterile filtration through 0.22 μm membrane filters.

##### 2.4.2.1 Quality assurance protocol

All procedures complied with USP 797 requirements for sterile compounding. Additional quality control measures included:

- Particulate matter analysis via light obscuration particle counting (USP < 788>) (19)
- Sterility validation by membrane filtration (USP < 71>)
- Endotoxin testing to ensure levels < 0.25 EU/ml
- Osmolality verification (280–310 mOsm/kg)
- pH stability monitoring (±0.1 units/24 h)

##### 2.4.2.2 Interference control

To eliminate potential optical interference from undissolved particulates, all solutions underwent dual filtration through 0.22 μm membranes. While complete elimination of microbubbles is theoretically unattainable, rigorous standardization of preparation and pipetting techniques minimized their formation. All measurements were conducted after thermal equilibrium (30-min stabilization) to eliminate transient signal artifacts. These comprehensive controls ensured that observed signal variations in the photometric detection system were exclusively attributable to calcium-phosphate precipitate formation rather than non-specific interference.

#### 2.4.3 Experimental design

The experimental procedure was conducted as follows: A double-layer beaker containing 60 ml of phosphorus-containing substrate was positioned on the electromagnetic stirrer operating at 1,500 rpm. Following system initialization and stabilization of both temperature and photometric signals, the titration protocol was initiated.

A sequential titration strategy was employed, beginning with high-concentration calcium preparations (45 mmol/L) to establish the precipitation threshold. Titration continued until the photometric signal decreased by 4 × 10^2^ Lux, indicating approach to the critical precipitation point. Subsequent refinement using low-concentration calcium preparations (5 mmol/L) was performed until visible precipitate formation was confirmed.

The experimental protocol incorporated the following standardized parameters:

- Dosing volume of the calcium preparation: 10 μl per addition
- Inter-dose interval: 10 min between successive calcium additions
- Termination criterion A: illuminance reduction ≥5 × 10^2^ Lux
- Termination criterion B: calcium concentration ≥30 mmol/L in reaction vessel

Notably, the upper calcium concentration limit (30 mmol/L) significantly exceeds the maximum clinical dosage of 10 mmol/L for parenteral nutrition (20), thereby establishing an appropriate safety margin for compatibility assessment while maintaining clinical relevance. This systematic approach ensured precise determination of calcium-phosphate compatibility limits while simulating realistic clinical formulation scenarios. The experimental design in this study should be understood as a structured multi-factor dynamic design under clinically relevant conditions, rather than as a simple static comparison of a limited number of fixed experimental combinations. Specifically, six calcium-phosphorus formulation combinations were evaluated across predefined levels of phosphorus concentration, pH, temperature, glucose, 18AA-II, and 18AA-V, as summarized in Table 2. Under each condition, calcium was added sequentially by titration to determine the precipitation threshold. Therefore, the 15,342 observations reported in this study represent cumulative dynamic measurement points generated during the transition from stability to precipitation across all tested conditions, rather than merely the number of unique static factor combinations.

To further clarify the structure of the dataset, this experimental design aimed to obtain multiple independent datasets from a single experiment using a dynamic monitoring approach. To ensure the relative independence of the data, a 10-min interval was applied after each calcium addition to allow the system to reach a relatively stable state. Subsequently, the presence or absence of precipitation was determined, and the real-time system parameters at that titration step—including calcium concentration, phosphorus concentration, pH, temperature, etc.—were recorded. Thereafter, the next titration was performed. In other words, each titration step altered the formulation composition and precipitation-risk state of the system, and the resulting observations were independent of one another.

#### 2.4.4 Statistical design of the study

Statistical analyses were performed using SPSS Statistics software (Version 26.0; IBM Corp., Armonk, NY, USA). Pearson's chi-square test was used to assess the association between calcium-phosphorus combination type and compatibility outcome. Subsequently, least absolute shrinkage and selection operator (LASSO) regression was applied for penalized variable selection to identify the key predictors of precipitation formation. Precipitation status (presence or absence) was defined as the binary dependent variable, and all candidate variables, including calcium concentration, phosphorus concentration, pH, temperature, glucose concentration, 18AA-II concentration, and 18AA-V concentration, were entered into the model as independent variables. The optimal penalty parameter (denoted as *K* in the software output) was determined from the coefficient path plot, and the minimum *K* value at which the standardized regression coefficients became stable was selected. LASSO regression enables variable selection while controlling model complexity, thereby improving model stability and interpretability.

To further assess the reliability of the LASSO findings, multivariable logistic regression was performed as a complementary analysis. Variables with *p* < 0.05 in univariable analysis were entered into the multivariable model. Model goodness-of-fit was assessed using the Hosmer–Lemeshow test, and multicollinearity was evaluated using variance inflation factors (VIFs). All statistical tests were two-sided, with the significance level set at α = 0.05. Odds ratios (ORs) and 95% confidence intervals (CIs) were calculated for statistically significant predictors.

### 2.5 Calcium-phosphorus preparation compatibility modeling

#### 2.5.1 Dataset description and data preprocessing

The experimental dataset was generated using the aqueous-phase dynamic precipitation monitoring system under controlled conditions, yielding 15,342 complete observations. The data were stratified by phosphorus substrate: KH_2_PO_4_ (*n* = 4,518), β-GP (*n* = 5,280), and FBP (*n* = 5,544).

A standardized preprocessing pipeline was applied to ensure data quality. Missing values: All experimental data were independently entered by two researchers and cross-validated. The experimental protocol was fully standardized, with fixed procedures for each condition, resulting in no missing fields in the dataset. Outliers: All instruments were calibrated before experiments, and operational stability was verified through preliminary runs. During formal experiments, real-time monitoring was performed; if any anomaly was detected (e.g., illuminance signal fluctuation, temperature deviation >0.5 °C, inconsistent stirring speed), the run was immediately terminated and repeated after correction. Consequently, unstable data were excluded at the acquisition stage and did not enter the final dataset. Interquartile range (IQR) analysis of the 15,342 retained observations detected no values beyond 3 × IQR, indicating good repeatability and stability of experimental execution. Finally, feature scaling was applied to normalize the data and ensure compatibility with the modeling algorithms.

#### 2.5.2 Model development

Predictive modeling was performed using IBM SPSS Modeler 18.0. Twelve initial models were constructed by applying four distinct algorithms—C5.1 decision tree, Chi-squared Automatic Interaction Detector (CHAID), support vector machine (SVM) with a radial basis function kernel, and a feed-forward artificial neural network (ANN)—to three datasets: the KH_2_PO_4_+CaCl_2_ combination, the KH_2_PO_4_+CaGlu combination, and the comprehensive six-formulation dataset.

Input features for model construction were limited to predictors identified as statistically significant (*p* < 0.05) in the prior multivariable logistic regression analysis. Using the Partition node, the dataset was randomly divided into a training set (70%) and a hold-out test set (30%), with a fixed random seed specified to ensure reproducibility. The C5.1 decision tree model was developed on the training set, and 10-fold cross-validation was performed within the training set to assess internal model stability and generalizability. Model performance was then evaluated on the hold-out test set using metrics including accuracy and the area under the receiver operating characteristic curve (AUC). By combining cross-validation within the training set with evaluation on a hold-out test set, this strategy improved the robustness of performance estimation and reduced the risk of overfitting.

#### 2.5.3 Model evaluation

Model performance was evaluated using the area under the receiver operating characteristic curve (AUC-ROC), complemented by overall accuracy. Models with discriminative capacity (AUC > 0.5) were retained for subsequent analysis. Final model selection employed a multi-criteria strategy that prioritized high discriminative performance (AUC) and clinical interpretability. Among these top-performing models, the one achieving the highest accuracy was selected for clinical deployment, ensuring a balance between predictive power and practical utility. To further assess generalizability, the final selected models were subsequently evaluated using an independent external validation set consisting of 100 clinical samples collected under realistic compounding conditions.

## 3 Results

### 3.1 Status of data analysis

**Figure 2:**
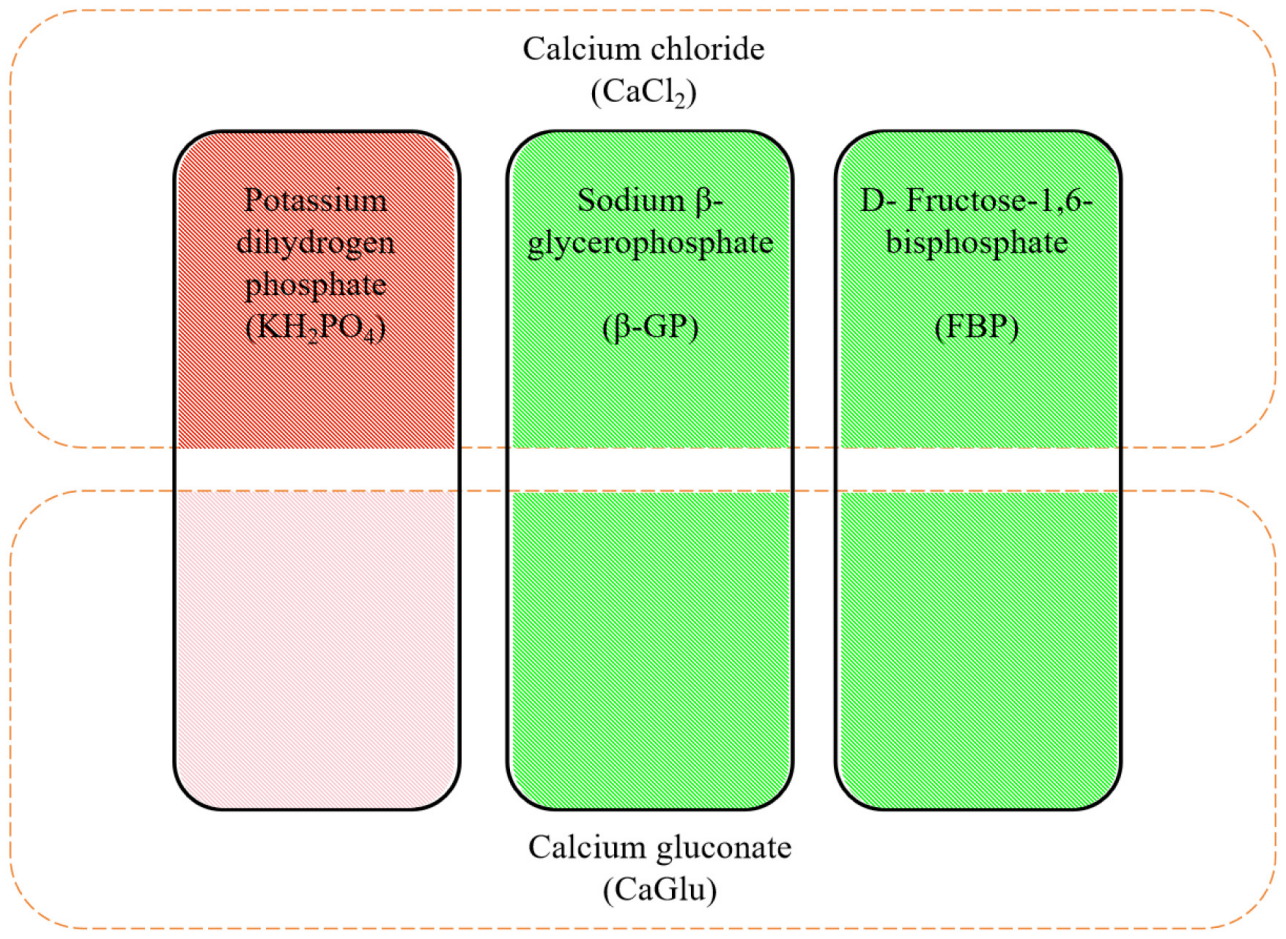
Compatibility of calcium and phosphorus combinations in parenteral nutrition solutions. Shaded green indicates combinations with no precipitation observed, red indicates a high risk of precipitation, and light red indicates a lower risk of precipitation.

Systematic evaluation of six calcium-phosphorus formulations under varied physicochemical conditions generated 15,342 complete observations, tracking the transition from stability to precipitation onset (Table 3). The distribution of precipitation events is shown in Figure 2.

**Table 3:** Data from experimental groups with different substrates.

Combination	Calcium concentration range (mmol/L)	Phosphorus concentration range (mmol/L)	pH	Temperature (°C)	Glucose (%)	18AA-II (%)	18AA-V (%)	Experimental group	Precipitation group
CaCl_2_+KH_2_PO_4_	0–30	0–20	6, 7, 8	20, 30	0, 5, 10	0, 8.5, 11.4	0, 3.2	2,234	461
CaGlu+KH_2_PO_4_	0–30	0–20	6, 7, 8	20, 30	0, 5, 10	0, 8.5, 11.4	0, 3.2	2,284	347
CaCl_2_+β-GP	0–30	0–150	6, 7, 8	20, 30	0, 5, 10	0, 8.5, 11.4	0, 3.2	2,640	None
CaGlu+β-GP	0–30	0–150	6, 7, 8	20, 30	0, 5, 10	0, 8.5, 11.4	0, 3.2	2,640	None
CaCl_2_+FBP	0–30	0–150	6, 7, 8	20, 30	0, 5, 10	0, 8.5, 11.4	0, 3.2	2,772	None
CaGlu+FBP	0–30	0–150	6, 7, 8	20, 30	0, 5, 10	0, 8.5, 11.4	0, 3.2	2,772	None

A critical finding was that the phosphate type dictated compatibility. All four combinations involving organic phosphates (β-GP and FBP) remained stable across all tested conditions with both calcium sources (CaCl_2_ and CaGlu). In contrast, the two inorganic phosphate (KH_2_PO_4_) combinations consistently exhibited precipitation risk. Consequently, subsequent analysis focused on these two incompatible pairs—CaCl_2_+KH_2_PO_4_ and CaGlu+KH_2_${\text{PO}}_{4}^{--}$ to enable a detailed mechanistic investigation.

### 3.2 Statistical analysis

**Figure 3:**
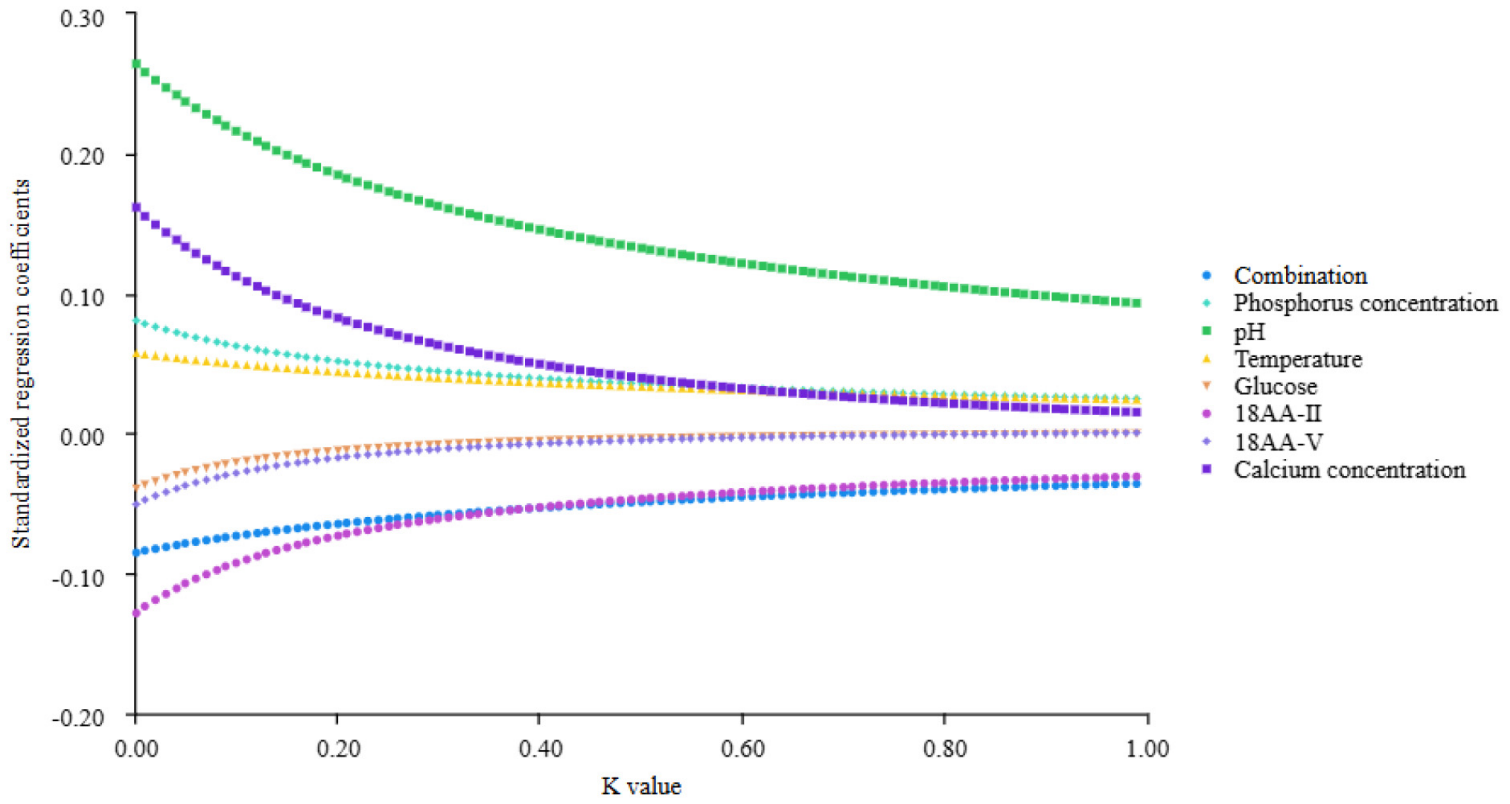
Lasso trajectory plot.

LASSO regression was used to assess the relative importance of candidate predictors of precipitation formation. When *K* = 0.01, the standardized regression coefficients of all variables became stable, and none of the coefficients was shrunk to zero, as shown in Figure 3. The coefficient directions indicated that calcium concentration, phosphorus concentration, pH, and temperature were positively associated with precipitation formation, whereas glucose concentration, 18AA-II, and 18AA-V were negatively associated with precipitation formation (Table 4). Based on the absolute values of the standardized regression coefficients, the relative importance of the variables was ranked as follows: pH > calcium concentration > 18AA-II > phosphorus concentration > temperature > 18AA-V > glucose concentration. The results of the multivariable logistic regression analysis were generally consistent with the LASSO findings in terms of the main predictors, identifying six statistically significant predictors of precipitation formation. Specifically, phosphorus concentration, pH, temperature, 18AA-II, 18AA-V, and calcium concentration were all statistically significant (*p* < 0.05), whereas glucose concentration was not (*p* > 0.05). Multicollinearity diagnostics showed that all variance inflation factors were < 5 and all tolerance values were > 0.2, indicating stable model estimation. Pearson's chi-square test further confirmed that calcium–phosphorus combination type was significantly associated with precipitation formation (χ^2^ = 22.783, *p* < 0.001). Overall, these statistical analyses identified seven important factors associated with precipitation risk, including six statistically significant predictors identified by multivariable logistic regression and one additional factor related to calcium–phosphorus combination type identified by chi-square analysis. Glucose concentration was not retained as a statistically significant predictor. All statistically significant factors were subsequently included in the construction of the predictive model.

**Table 4:** Results of statistical analysis.

Influence factor	Lasso regression analysis	Logistic regression analysis				
	*R*	B	*P*-value	OR	95% CI for OR	
					Lower	Upper
Phosphorus concentration	0.078	0.036	0.000	1.036	1.022	1.051
pH	0.250	0.882	0.000	2.416	2.127	2.745
Temperature	0.057	0.032	0.000	1.032	1.016	1.049
Glucose	−0.036	−2.514	0.073	0.081	0.005	1.260
18AA-II	−0.121	−7.181	0.000	0.001	0.000	0.008
18AA-V	−0.047	−11.601	0.006	0.000	0.000	0.034
Calcium concentration	0.140	0.057	0.000	1.059	1.044	1.074
Constant		−8.837	0.000	0.000		

### 3.3 Analysis of influencing factors

#### 3.3.1 pH-dependent precipitation behavior

**Figure 4:**
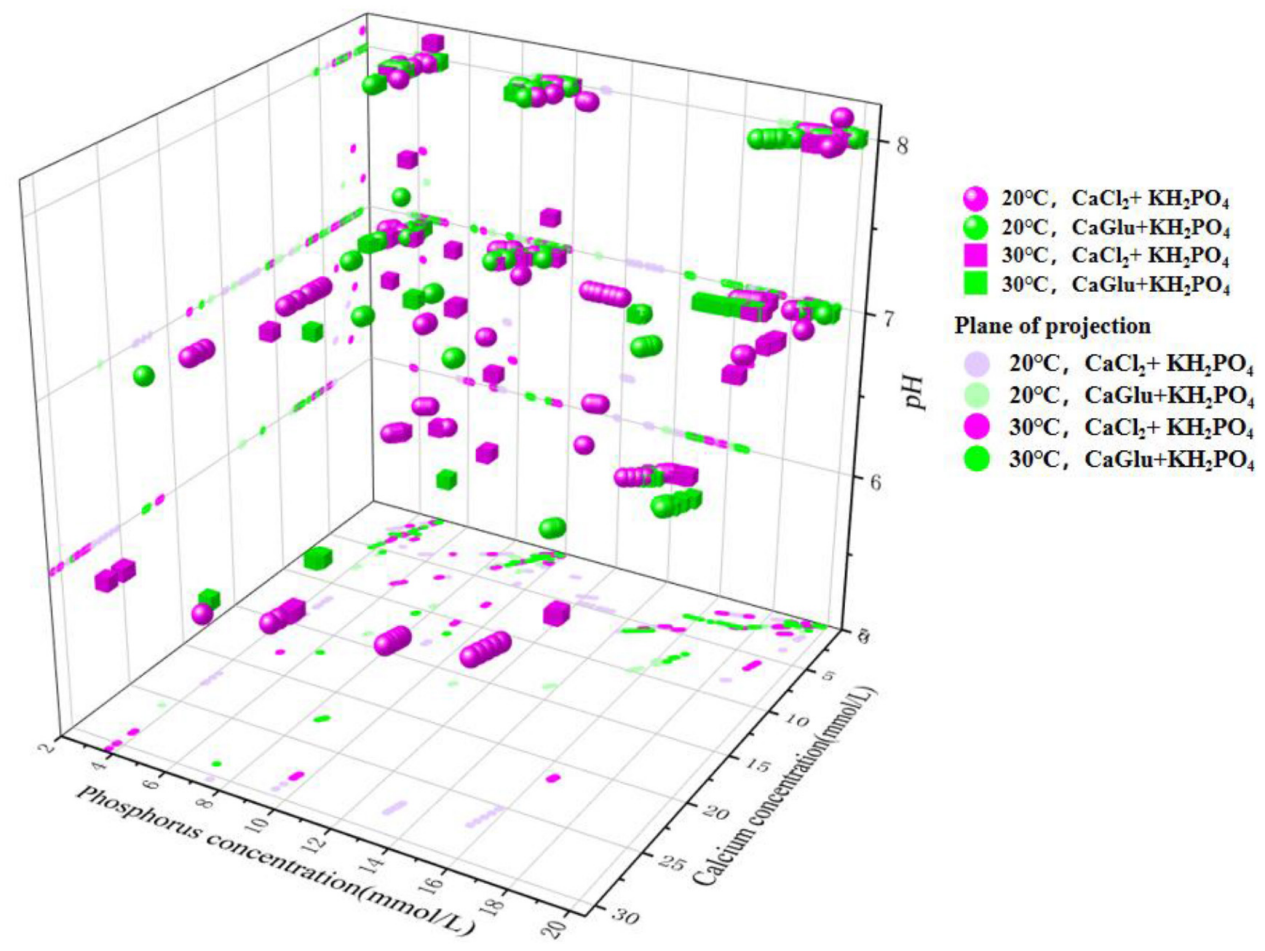
The three-dimensional distribution of calcium-phosphorus precipitation for CaCl_2_+KH_2_PO_4_ and CaGlu+KH_2_PO_4_ combinations under different experimental conditions.

**Figure 5:**
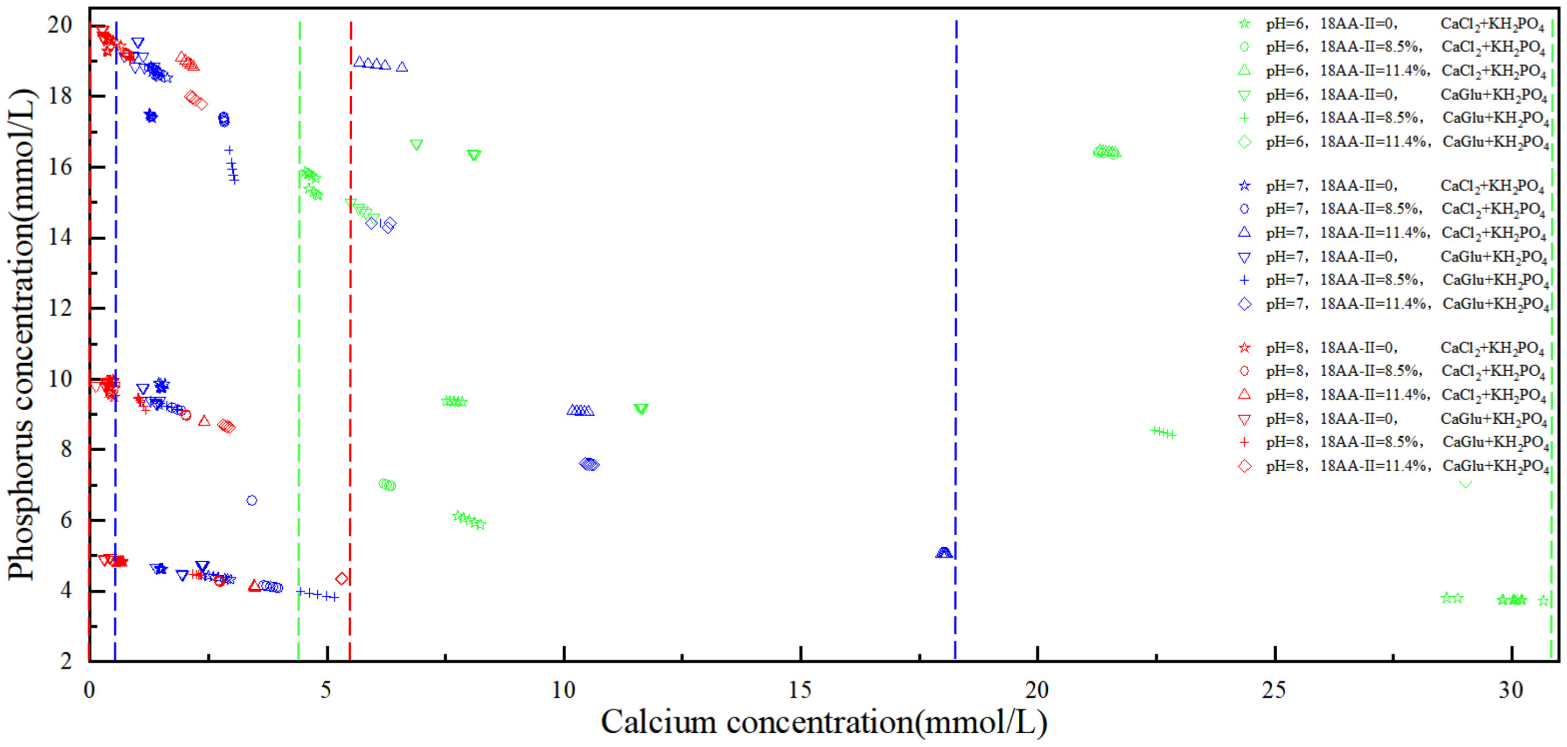
Scatter plot showing the effect of pH on the compatibility of CaCl_2_+KH_2_PO_4_ and CaGlu+KH_2_PO_4_ combinations.

Multivariate visualization revealed pronounced pH-dependent precipitation dynamics (Figure 4). As pH increased from 6 to 8, the threshold concentrations of calcium and phosphorus required to initiate precipitation substantially decreased (Figure 5). Concomitantly, precipitation frequency increased by 2.2-fold, with precipitation loci becoming spatially concentrated in regions of lower calcium concentration. A comparative analysis between the two inorganic phosphate combinations showed that CaCl_2_+KH_2_PO_4_ was significantly more prone to precipitation than CaGlu+KH_2_PO_4_ under identical pH conditions, and its precipitation distribution was shifted toward lower calcium concentrations (*p* < 0.01).

#### 3.3.2 Temperature acceleration effects

**Figure 6:**
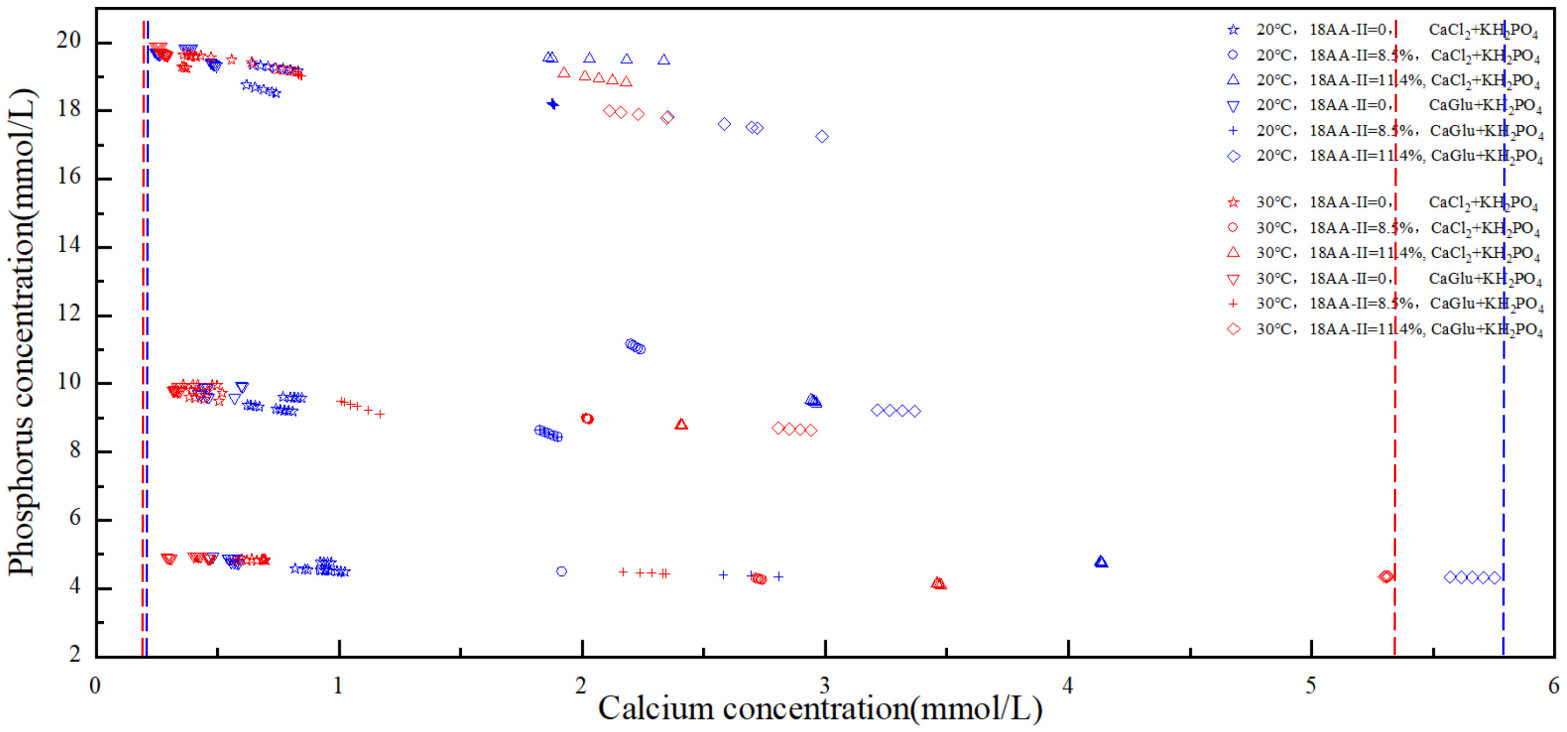
Scatter plot showing the effect of temperature on the compatibility of CaCl_2_+KH_2_PO_4_ and CaGlu+KH_2_PO_4_ combinations.

Temperature elevation from 20 °C to 30 °C significantly accelerated precipitation kinetics (Figure 6), reducing precipitation thresholds by 56 ± 3% and increasing precipitation frequency by 18 ± 5%. Spatial analysis confirmed accelerated nucleation under elevated temperatures (Figure 4, XY projections). The precipitation threshold was reduced by 77% for CaCl_2_+KH_2_PO_4_, whereas only a 35% reduction was observed for CaGlu+KH_2_PO_4_.

#### 3.3.3 Formulation-specific compatibility

**Figure 7:**
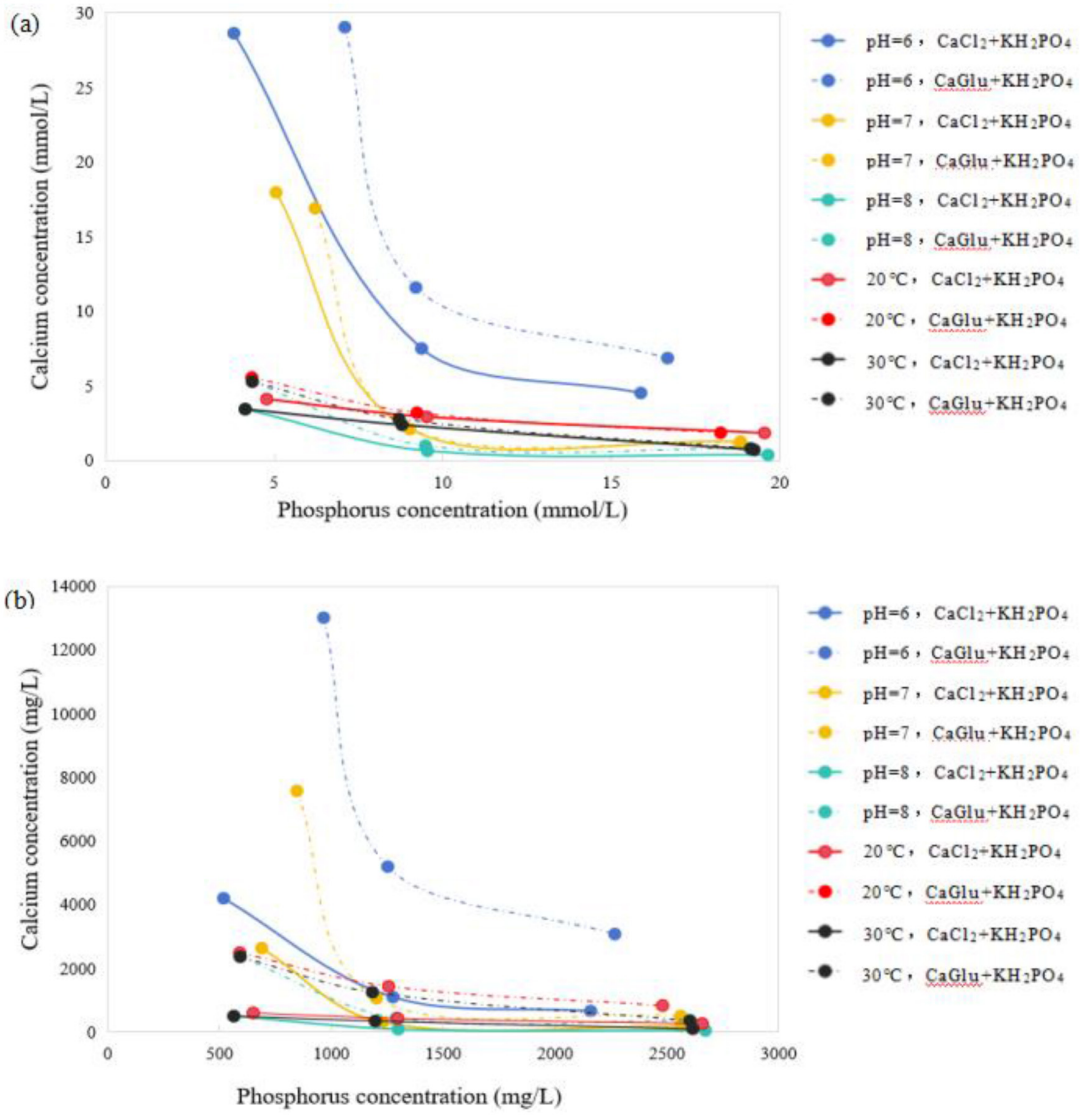
Effect of calcium and phosphorus type and concentration on calcium and phosphorus compatibility. **(a)** Cross-section of calcium and phosphorus precipitation in mmol/L. **(b)** Cross-section of calcium and phosphorus precipitation in mg/L.

Concentration-dependent analysis established critical incompatibility thresholds for the inorganic combinations (Figure 7). The CaCl_2_+KH_2_PO_4_ system demonstrated the greatest tendency for precipitation, requiring a lower calcium concentration compared to CaGlu+KH_2_PO_4_ at the same phosphorus level. The mass concentration representation (Figure 7b), which offers enhanced clinical translatability, clearly demonstrates the superior safety profile of calcium gluconate formulations.

#### 3.3.4 Amino acid-mediated stabilization

**Figure 8:**
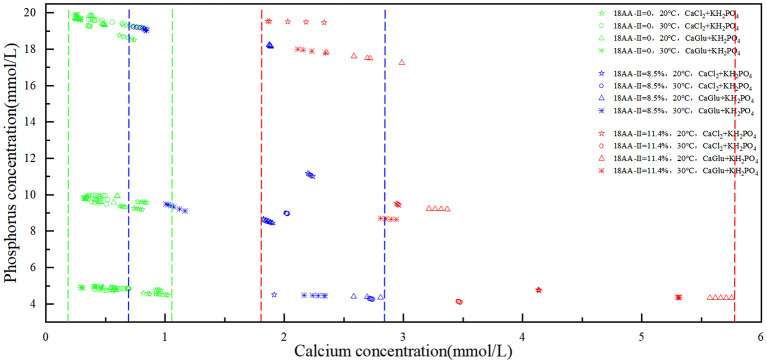
Scatter plot showing the effect of 18AA-II on the compatibility of CaCl_2_+KH_2_PO_4_ and CaGlu+KH_2_PO_4_ combinations.

**Figure 9:**
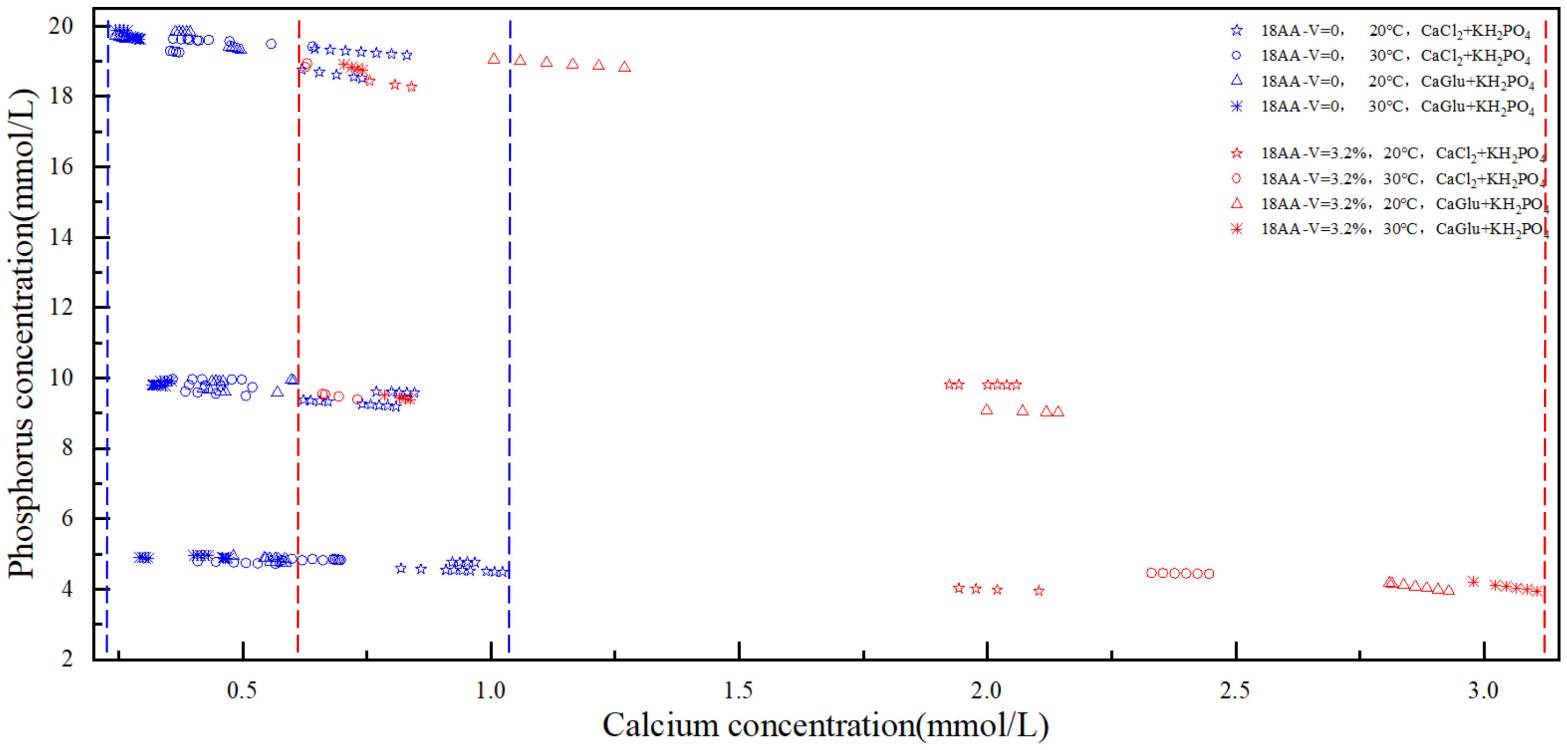
Scatter plot showing the effect of 18AA-V on the compatibility of CaCl_2_+KH_2_PO_4_ and CaGlu+KH_2_PO_4_ combinations.

Both 18AA-II and 18AA-V demonstrated concentration-dependent inhibition of precipitation (Figures 8 and 9). Incremental increases in 18AA-II (0% → 11.4%) and 18AA-V (0% → 3.2%) elevated precipitation thresholds by 7.8-fold and 1.9-fold, respectively. Throughout this concentration range, the CaGlu+KH_2_PO_4_ combination maintained superior compatibility, with its precipitation loci consistently positioned at significantly higher calcium concentrations than CaCl_2_+KH_2_PO_4_ (*p* < 0.001).

This systematic analysis establishes pH, temperature, and calcium/phosphorus concentrations as key risk factors for precipitation, while suggesting that 18AA-II and 18AA-V may be important inhibitory agents. The consistently superior compatibility profile of the CaGlu+KH_2_PO_4_ formulation provides a critical foundation for optimizing the safety of parenteral nutrition regimens.

### 3.4 Predictive modeling and clinical implementation

#### 3.4.1 Model performance and selection

Predictive models were developed using six clinically significant variables (temperature, pH, calcium concentration, phosphorus concentration, 18AA-V, and 18AA-II) identified through prior statistical analysis. As summarized in Table 5, all classifiers demonstrated discriminative capacity with AUC values exceeding 0.5. The ensemble model integrating six calcium-phosphate combinations achieved superior predictive performance, with the C5.1 algorithm exhibiting optimal metrics (accuracy: 98.5%; AUC: 0.985). Notably, four of the six model configurations surpassed the performance of single-combination predictors (CaCl_2_+KH_2_PO_4_ and CaGlu+KH_2_PO_4_), with all ensemble models maintaining >90% accuracy and AUC values >0.85, confirming robust predictive capability across formulation scenarios.

**Table 5:** Performance of models.

Modeler	CaCl_2_+KH_2_PO_4_		CaGlu+KH_2_PO_4_		Six combinations	
	Overall accuracy (%)	AUC	Overall accuracy (%)	AUC	Overall accuracy (%)	AUC
C5.1	95.792	0.976	95.403	0.981	98.494	0.985
CHAID	83.259	0.882	86.340	0.900	94.733	0.931
ANN	85.094	0.877	85.552	0.852	94.733	0.888
SVM	80.931	0.883	86.821	0.897	95.183	0.939

#### 3.4.2 Internal validation

Internal validation showed that the C5.1 decision tree model achieved good predictive performance and stability. The 10-fold cross-validation accuracy was 96.7%, indicating robust performance during internal validation. Evaluation on the hold-out test set yielded an accuracy of 94.9%. The small difference between the cross-validation and test-set accuracies suggests that the model had good generalizability and no evidence of substantial overfitting.

#### 3.4.3 External validation

Model robustness was further evaluated using 100 independently prepared validation samples representing clinically relevant compounding conditions. These samples were not obtained directly from existing clinical prescriptions; instead, they were prepared from commercial injectable formulations according to concentration ranges commonly used in parenteral nutrition practice. This strategy was adopted because lipid emulsions are routinely included in clinical parenteral nutrition prescriptions, whereas the present study specifically focused on calcium-phosphorus compatibility and precipitation risk in the aqueous phase. Direct use of complete lipid-containing formulations would have complicated the observation and assessment of aqueous precipitation. Therefore, 100 external validation samples were independently prepared within clinically relevant formulation ranges to evaluate model performance under simulated real-world compounding conditions. The C5.1-based predictors demonstrated sustained performance, with accuracy rates of 83%, 86%, and 95% for the CaCl_2_+KH_2_PO_4_, CaGlu+KH_2_PO_4_, and six-combination models, respectively (Table 6). These findings support the reliability of the models under clinically relevant conditions, and the superior performance of the six-combination model further highlights its stronger generalization capability for more complex formulation assessments.

**Table 6:** Results of model validation.

Combination	Test dataset size	Validation match cases	Accuracy rate (%)
CaCl_2_+KH_2_PO_4_	100	83	83
CaGlu+KH_2_PO_4_	100	86	86
Six combinations	100	95	95

#### 3.4.4 Clinical translation platform

**Figure 10:**
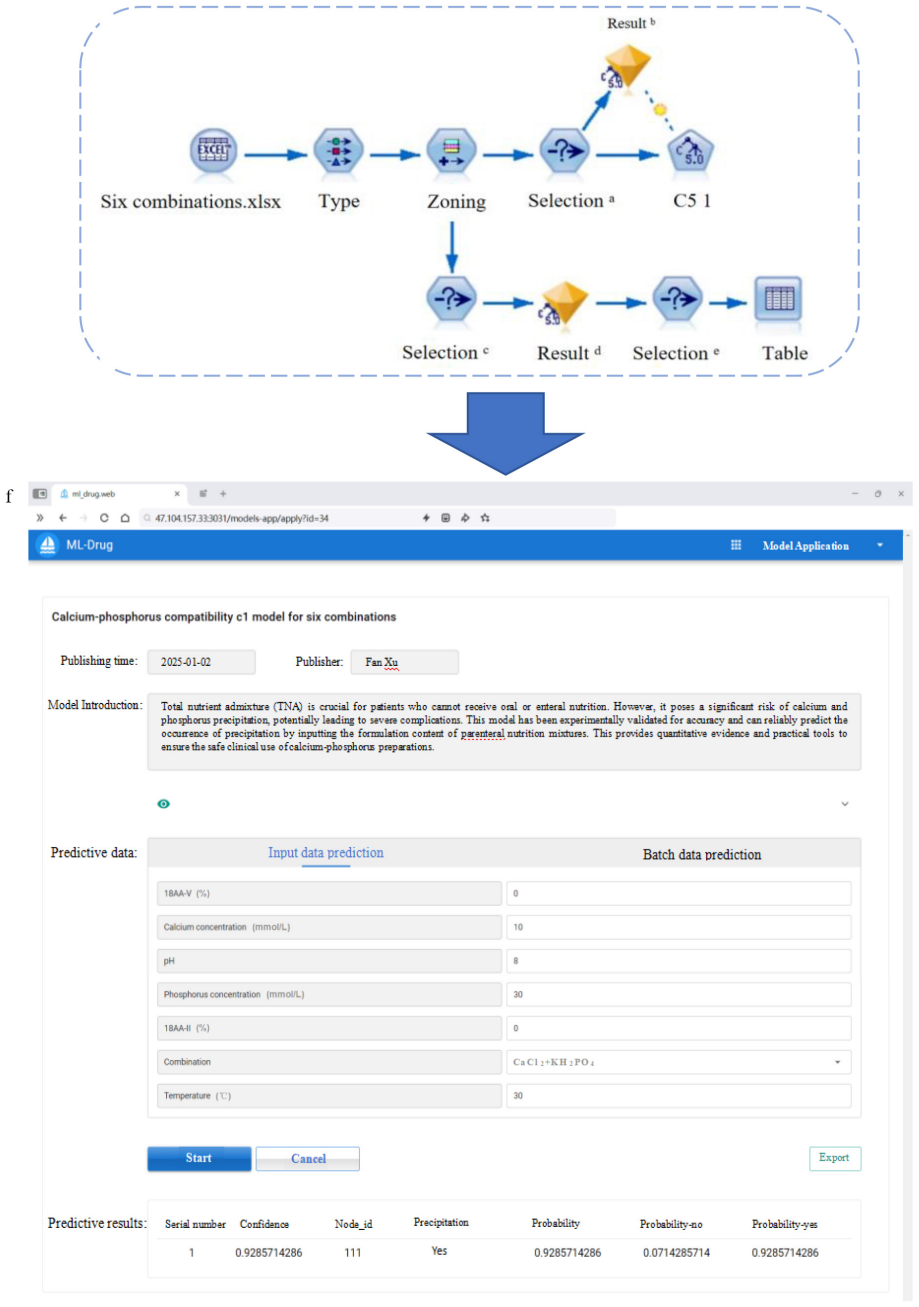
Overview of model development, validation, and application interface. (a) Selection of training set for model development. (b) Construction of the C5.1 decision tree model. (c) Determination of test set size. (d) Model validation results. (e) Matching of predicted results with test set to obtain equivalent sample size. (f) Screenshot of the ML-Drug platform interface: the “Predictive data” panel displays example input parameters (e.g., 18AA-V, calcium concentration, pH, phosphorus concentration, 18AA-II, temperature, and calcium–phosphorus combination type), with a toggle at the top to switch between single–entry and batch data prediction. After user input, clicking the “Start” button generates the “Predictive results” panel, which shows precipitation risk (“Yes”/”No”), confidence level, and probability scores for both classes.

To facilitate clinical implementation, the optimized C5.1 algorithm was deployed on the Machine Learning-Drug (ML-Drug) platform, enabling web-based prediction of calcium-phosphate compatibility. As demonstrated in Figure 10, the interface supports both single-point predictions through parameter input and batch processing via standardized templates. This digital solution provides healthcare professionals with immediate risk assessment capabilities, effectively bridging analytical findings with clinical decision-making for parenteral nutrition safety optimization. To further demonstrate the translational value of the model, we constructed an illustrative clinical scenario to show the decision-support role of the ML-Drug platform in optimizing parenteral nutrition (PN) prescriptions. For example, when a clinician prepares a PN prescription for a patient and needs to determine whether the formulation carries a risk of precipitation, the relevant prescription parameters (e.g., calcium and phosphate concentrations, pH, temperature, amino acid composition, and other key formulation variables) can be entered into the ML-Drug platform. The system then automatically predicts the likelihood of calcium-phosphate precipitation. If a precipitation risk is identified, the platform provides feedback to guide adjustment of the prescription composition. The revised formulation can subsequently be re-entered into the platform for re-evaluation. Through this iterative process, an optimized and clinically safe PN formulation with acceptable compatibility can be obtained. In this way, PN compatibility assessment can move from empirical decision-making toward a more data-assisted and quantitative approach.

The transferability of the platform is supported by the incorporation of clinically common calcium-phosphate admixture combinations and comprehensive consideration of the major factors affecting their safety, including calcium source, phosphate source, concentration range, pH, temperature, and other compatible components such as glucose and amino acid formulations, rather than relying on precipitation thresholds derived from a single influencing factor. Therefore, when applied to conventional aqueous parenteral nutrition admixture systems, the model can provide data-driven predictions of precipitation risk, provided that the corresponding formulation and environmental parameters are available. However, the applicability of the model remains limited by the range of conditions represented in the training dataset. For systems outside the variable ranges investigated in this study or involving different types of preparations, further external validation and model updating would be required prior to direct application.

At present, the platform has recorded just over 200 visits, suggesting that its real-world dissemination remains at an early stage. Wider promotion and broader clinical adoption will therefore be needed to further realize its translational potential.

## 4 Discussion

### 4.1 Methodological innovation and precipitation mechanisms

Calcium-phosphate precipitation is one of the most common chemical incompatibilities in Total Parenteral Nutrition (TPN) manufacturing (21). Our study addresses this challenge through the development of a dynamic monitoring system based on photoresistance principles, enabling real-time observation of precipitation kinetics in aqueous phases. Under the tested conditions, both calcium forms (CaCl_2_ and CaGlu) remained compatible with the organic phosphorus compounds (β-GP and FBP), with no precipitation observed in these combinations (Table 3). These findings support the compatibility of organic phosphate formulations within the examined experimental range. One possible explanation is that the molecular structure of organic phosphate esters alters their interaction with calcium ions, thereby reducing the tendency to form insoluble precipitates. For example, the covalent linkage between phosphate groups and the molecular backbone, together with potential steric and coordination effects, may contribute to improved solubility. However, these mechanistic interpretations were not directly tested in the present study and should therefore be regarded as hypotheses rather than demonstrated effects. Our findings are consistent with previous reports by Anderson and MacKay (22) and MacKay and Anderson (12) regarding the favorable compatibility profiles of organic phosphates, while providing more detailed quantitative information across formulation combinations and experimental conditions.

### 4.2 Formulation-dependent compatibility profiles

The positive association between pH elevation and precipitation risk observed in this study is consistent with established principles of calcium phosphate solubility (23). Beyond confirming the critical role of pH in determining compatibility limits, our data also revealed clear formulation-dependent differences. Under comparable conditions, CaCl_2_ exhibited a higher precipitation propensity than CaGlu, which aligns with Bouchoud's (14) observations in neonatal formulations. A possible explanation for this difference is that the two calcium salts may exhibit different dissociation behaviors in solution, which in turn could influence free calcium activity and the rate at which the system approaches supersaturation. However, this interpretation remains speculative, as free-ion activity and related physicochemical parameters were not directly measured in the present study.

### 4.3 Environmental and compositional influences

Temperature elevation (20 °C to 30 °C) significantly accelerated precipitation kinetics, corroborating Prinzivalli's reports of reduced compatibility limits under elevated temperatures (16). This thermal enhancement likely reflects increased ion mobility and dissociation constants, promoting nucleation events. Furthermore, our expanded safety ranges compared to historical data (24) probably derive from the protective effects of specific amino acid formulations (18AA-II, 18AA-V), whose buffering capacities and molecular interactions provide additional stabilization beyond pH modulation alone. The negligible impact of glucose concentration aligns with Ribeiro's et al. (25) findings, suggesting that maintained pH stability (6–8 range) in final admixtures minimizes its direct chemical influence.

The strong correlation observed between 18AA-II/18AA-V and reduced precipitation risk requires direct validation of the underlying mechanisms. Based on current knowledge, we propose the following hypotheses: (1) Buffering effect: Amino acid formulations may inhibit calcium–phosphate nucleation by maintaining a lower pH, an effect that extends beyond the influence of pH alone; (2) Chelation: Amino acid constituents (e.g., cysteine, histidine) may form soluble complexes with calcium ions via their side–chain groups, thereby reducing free calcium activity; (3) Steric hindrance: Amino acid molecules may adsorb onto the surfaces of nascent crystals, impeding crystal growth. However, none of these mechanisms have been directly demonstrated in the present study and should be regarded as data–based hypotheses rather than demonstrated effects. Future studies should employ experimental designs such as individually adding specific amino acid components to confirm causality.

### 4.4 Methodological considerations and clinical translation

TNA typically consist of both an aqueous phase and a lipid phase, and their safety evaluation requires independent assessment of each component. The monitoring technique employed in this study is applicable only to the aqueous phase. This limitation arises because the turbidity and light-scattering effects of lipid emulsions can significantly interfere with optical detection methods, thereby restricting their application in lipid-containing systems. Accordingly, the calcium–phosphorus compatibility data and predictive models established in this study are currently applicable to: (1) the overall safety evaluation of lipid-free TNA, and (2) the assessment of the aqueous phase in lipid-containing TNA formulations.

A comprehensive safety evaluation of TNA should be conducted in two stages. First, the aqueous phase should be investigated to establish a compatibility safety range, which constitutes the focus of the present study. Second, the safety of the lipid phase–particularly the presence of large lipid droplets following the incorporation of lipid emulsions–should be systematically evaluated, which will be addressed in future work. Overall, for TNA formulations, satisfactory safety can only be ensured when both the aqueous and lipid phases meet their respective safety criteria. The consistent use of molar concentrations ensures precise stoichiometric relationships, with experimental ranges (calcium: ≤ 20 mmol/L; organic phosphorus: ≤ 150 mmol/L) covering clinical requirements (20–40 mmol phosphorus/day) (26) while respecting identified compatibility limits.

The added value of this quantitative framework lies not only in determining whether precipitation occurs, but also in defining how precipitation thresholds shift under clinically relevant changes in pH, temperature, calcium concentration, phosphorus concentration, and amino acid composition. Unlike traditional *K*-value calculations or empirical compatibility charts, which mainly provide static reference limits under restricted conditions, our approach generates dynamic threshold information, quantifies the effect sizes of multiple predictors, and reveals formulation-specific response patterns. In addition, the C5.1-based model enables individualized risk prediction for a given formulation by integrating multiple variables simultaneously, thereby extending calcium-phosphorus compatibility assessment from static reference guidance to data-driven decision support. The web-based ML-Drug platform further facilitates clinical translation of these quantitative findings. It should be noted that, although the predictive model developed in this study achieved high accuracy in internal validation, the experimental data were generated through a sequential titration process. Consequently, some residual correlation may still exist among consecutive observations within the same experimental condition, and this possibility cannot be completely excluded. Such potential correlation may have partially contributed to the high internal accuracy and AUC values observed in the present study.

Nevertheless, the dynamic titration monitoring strategy enabled systematic coverage of multi-factor experimental conditions and efficient acquisition of a large-scale compatibility dataset under clinically relevant settings. Furthermore, the sustained predictive performance observed during external validation suggests that the model performance was not solely dependent on sequential similarity within the training data. Therefore, although the present approach has methodological limitations, it may still provide a useful framework for preliminary risk assessment and compatibility evaluation in clinical parenteral nutrition compounding.

## 5 Conclusions

This study systematically investigated the critical challenge of calcium-phosphorus compatibility in parenteral nutrition using an integrated experimental and computational approach. The following key findings were obtained, establishing a comprehensive application for compatibility assessment and prediction.

First, a systematic compatibility profile was established for six clinically used calcium-phosphorus formulation combinations under clinically relevant conditions. Using a rigorously validated real-time precipitation monitoring system, 15,342 high-quality observations were generated across the tested combinations. The results showed that organic phosphate formulations remained stable with all tested calcium sources, whereas inorganic phosphate combinations were associated with precipitation risk. These findings provide a quantitative basis for compatibility assessment and support safer parenteral nutrition formulation decisions.

Second, this study generated several new quantitative insights into the two precipitation-prone combinations, CaCl_2_+KH_2_PO_4_ and CaGlu+KH_2_PO_4_. Specifically, it quantified shifts in precipitation thresholds under different pH and temperature conditions, measured the effect sizes of key predictors through multivariable analysis, identified the concentration-dependent protective effects of 18AA-II and 18AA-V, and demonstrated formulation-specific differences in risk sensitivity between calcium chloride and calcium gluconate systems. Because compatibility is determined by multiple interacting factors, safe conditions cannot be reliably summarized by a single generalized statement. Accordingly, a predictive model was established to facilitate compatibility assessment across different formulations and experimental conditions. Beyond conventional static compatibility references, the C5.1-based framework integrates these variables to provide individualized prediction of precipitation risk, while its deployment on a web-based platform supports real-time clinical decision-making in parenteral nutrition formulation.

In summary, this work provides an integrated experimental and computational framework for addressing calcium-phosphorus compatibility challenges, encompassing mechanistic exploration, quantitative risk prediction, and practical implementation tools. The established methodology and predictive models offer a scientific foundation and practical reference for optimizing parenteral nutrition safety. It should be noted that the external validation sample size (*n* = 100) is limited, and larger multi-center validation studies are needed to further confirm the generalizability of the models.

## Data Availability

The datasets used and/or analysed during the current study are available from the corresponding author on reasonable request. Requests to access these datasets should be directed to Fan Xu, xu_fan@126.com.
